# Building successful and sustainable academic health science partnerships: exploring perspectives of hospital leaders

**Published:** 2019-03-13

**Authors:** Sarah DeBoer, Jamie Dockx, Chris Lam, Shabdit Shah, Gillian Young, Martine Quesnel, Stella Ng, Brenda Mori

**Affiliations:** 1 Department of Physical Therapy, University of Toronto, Ontario, Canada; 2 Department of Speech-Language Pathology, University of Toronto, Ontario, Canada; 3 Centre for Faculty Development, University of Toronto, Ontario, Canada

## Abstract

**Background:**

Clinical work-based internships form a key component of health professions education. Integral to these internships, academic health science partnerships (AHSPs) exist between universities and teaching hospitals. Our qualitative descriptive study explored the perspectives of hospital leadership on AHSPs: what they are composed of, and the facilitators and barriers to establishing and sustaining these partnerships.

**Methods:**

Fifteen individuals in a variety of hospital leadership positions were purposively sampled to participate in face-to-face interviews, after which a thematic analysis was conducted.

**Results:**

Participants reported that healthcare and hospital infrastructure shapes and constrains the implementation of clinical education. The strength of the hospitals’ relationship with the medical profession facilitated the partnership, however other health professions’ partnerships were viewed less favourably. Participants emphasized the value of hospital leaders prioritizing education. Further, our findings highlighted that communication, collaboration, and involvement are considered as both facilitators and barriers to active engagement. Lastly, opportunities stemming from the partnership were identified as research, current best practice, improved patient care, and career development.

**Conclusion:**

Our study found that AHSPs involve the drive of the university and hospitals to gain valued capital, or opportunities. Reciprocal communication, collaboration, and involvement are modifiable components that are integral to optimizing AHSPs.

## Introduction

Health professional training programs generally require clinical, workplace-based education prior to degree attainment.^1^ Much of this clinical education occurs in teaching hospitals,^2^ and therefore academic partnerships between universities and teaching hospitals are crucial to sustaining and advancing clinical education. Throughout this paper, university refers to an academic institution that provides education programs in the pursuit of a degree, masters or doctoral studies, where we are specifically focusing on health professional education programs.^3^

Hospitals that partner with universities to help teach the next generation of healthcare providers are called Academic Health Science Centres (AHSCs). Teaching hospitals have a long history of being indispensable to medical education, however the term academic medical center didn’t come into effect until after the second world war.^4^ Regardless of whether the hospital was owned privately by the medical schools, or publicly owned with an agreement of affiliation to the medical school, their independent success depended on their collaboration.^4^ AHSCs and universities share a common mandate of education and research, which has historically led to a strong and sustainable partnership, despite facing external pressures.^4^^,^^5^

After the Vietnam war, AHSP were challenged by urban decay in major cities, resulting in community strains, including the economic burden of serving uninsured and indigent patients. This led to the shift of academic health centers from charitable institutions to corporate vendors of service. Further, AHSC have been challenged by the clinical competition for private patients and thus private funding.^4^

Currently, increased student enrollment and a changing healthcare system in Canada have made these partnerships more complex, with implications for future clinical education. For example, the introduction of a new funding model by the Ministry of Health and Long Term Care restructured the use of healthcare resources in Ontario.^6^

Healthcare professions in the province have responded differently to these changes in healthcare funding despite the Academic Health Science Partnership (AHSP) being governed under one agreement. For example, the medical profession has adopted the use of an Alternative Funding Plan to ensure physicians are personally compensated for their participation in clinical education.^7^ In contrast, the physical therapy profession allocates funding to hospital organizations rather than directly reimbursing supervising healthcare professionals. There is no regulation on the specific use of these funds within physical therapy, resulting in an inconsistent utilization among hospitals.^8^

Continual changes in healthcare funding and the unique allocation of funding across healthcare professions contribute to the complexities of partnerships between universities and AHSCs. This complexity has contributed to difficulty in obtaining sufficient clinical education placements for students across health professions.^9^^,^^10^ In order to advance clinical education, it will be important to respond and adapt to the changing healthcare environment. Research on AHSPs has primarily focused on gaining clinical instructor and student perspectives to provide insight into the benefits and challenges of clinical education.^11^^–^^16^ However, participating in clinical education and partnering with universities is often dependent on senior leadership levels within hospitals.^2^^,^^17^

Hospital leaders are directly involved in the decisions concerning allocation of resources and engagement in clinical education.^2^ A survey identified that leadership staff value the opportunity to provide continued clinical education for hospital staff, carry out the mission statement of the organization, fortify the hospital’s reputation, and fulfill their responsibility as healthcare professionals to develop future clinicians.^2^ This research is a starting point for more in-depth research to inform strategies to sustain and advance AHSPs in an increasingly complex healthcare system. Therefore, this study aims to gain an in-depth understanding of the experiences of hospital leaders within AHSPs, with a focus on Physical Therapy. Our primary goal is to contribute knowledge and understanding to support symbiotic partnerships both locally and beyond.

## Methods

### Study design

Our study used a qualitative descriptive approach that allowed the research team to develop an understanding of hospital leaders’ experiences and perspectives in relation to AHSPs.

### Participants

Individuals were eligible to participate in our study if they held at least one of the following positions within an AHSC: Centre Coordinator of Clinical Education (CCCE), Professional Practice Lead (PPL), University Partnership in Academic Rehabilitation (UPAR) Committee Member, Director or Vice President (VP) of Education, Director or Vice President (VP) of Practice, or Chief Executive Officer (CEO). Further, we required participants to be employed at a hospital with which the university holds an AHSP, to hold an appointment within the university, and to consent to participate.

We used a maximum variation purposive sampling technique to recruit potential study participants.^18^ We identified a list of 130 potential participants in leadership positions from university-affiliated hospitals. We purposively sampled fifteen individuals based on their level of leadership, engagement with education as an institution, engagement with education in the field of Physical Therapy, status as a full versus community affiliated hospital, and gender. Ultimately, we aimed for three participants in each of the five leadership levels across the twenty-six fully or community affiliated hospitals to ensure heterogeneity in the perspectives capture by our study. Fully affiliated hospitals have a set commitment to clinical education opportunities outlined in an affiliation agreement between the university and hospital, while community teaching hospitals have no strict commitment, but are still considered an affiliate by the university.^19^

An email was sent to potential participants inviting them to be interviewed for the study, as well as outlining the purpose of the study and what would be required of their participation. If individuals responded indicating they wished to participate, a follow up email with a copy of a consent form and demographics questionnaire was sent to schedule a date and time for an interview.

### Data collection

We conducted fourteen semi-structured face-to-face interviews and one telephone interview with individuals who agreed to participate in the study. Two researchers attended each interview - one researcher who had undergone interview training conducted the interviews while the other facilitated the session by audio recording and taking field notes. We used a semi-structured interview guide (Appendix A). The guide was piloted and audio-taped with two individuals who both had knowledge of the academic and hospital settings. After piloting the interview guide, we made minor changes to the question structure, probing questions, interview flow, and overall interview length. The revised guide was used for all participants and involved open-ended questions, with a set of probing questions, to elicit the perspectives of hospital leaders on the components, facilitators, and barriers to a successful partnership.

### Data analysis

We used an inductive thematic analysis approach.^19^^,^^20^ The interviews were transcribed verbatim with all identifying information pertaining to participants or place of employment removed. Each transcript was initially reviewed twice - once by a team member present at the interview and once by a team member who was not. The coding process involved using a pen-and-paper method to highlight relevant meaning units in relation to our research question. Each meaning unit was identified, recorded, and numbered in a master list corresponding to that transcript. Common quotes were then grouped together by codes – labels for the meaning units – which were organized in a code book. Themes were established through the grouping, comparing, and contrasting of these codes into broader categories with a thematic map that considered relationships between themes as well as comprehensive coverage of the data extracted. The research was approved by and conducted in accordance with the Research Ethics Board at the University of Toronto.

## Results

Our study involved interviewing fifteen individuals, ranging in age from 37-60 years. Participants held a leadership position for an average of five years. Of the fifteen individuals interviewed, eight participants were associated with full-affiliated hospitals and seven with community-affiliated hospitals. Twelve woman and three men were interviewed who represented six different professional backgrounds including: medicine, physical therapy, occupational therapy, nursing, psychology, and health administration.

Our analysis generated the following six dimensions of influence in AHSPs: current infrastructure in Health Science Education, the relationship and model of medical education delivery in relation to other professions, values and perceptions espoused by hospitals, engagement between the hospital and the university, individuals’ engagement, and reciprocal opportunities.

### Current infrastructure in health science education

Participants discussed the role of physical and organizational structure in the operation of clinical education through AHSPs. For example, entering into “a formal affiliate agreement,” participant seven noted, “sets out mutual expectations and responsibilities, provides clarity to the partnership” (P7).

Yet, despite knowledge of these expectations and responsibilities, participants indicated that hospital policies and procedures could also limit hospital participation in clinical education. Specifically, policy regarding patient care was identified as a limitation to having the time to teach students: “Your time is absolutely measured, and you have dictated to you that in an eight-hour day you have to be providing X number of patient-care minutes to this many patients” (P4).

In addition to hospital policies, participants identified that resources afforded hospital participation in clinical education. Teaching students in the clinical environment requires supportive resources that are not always available for student learners:

*[We have to] make difficult decisions about how to balance budget [and] how to make cuts … it becomes more and more difficult every year and some of the things that end up suffering, as a result of it, are the teaching and research side.* (P10)

### The relationship and model of medical education delivery in relation to other professions

Participants identified that the strong and longstanding association hospitals have with medicine, influences the dynamics of the partnership, while identifying other health professions as more of an adjunct to the AHSPs. “There’s a stronger partnership, I would say, with the medical [profession] than there is with any of the other faculties” (P3). Participants also spoke about the communication within the partnership, stating that “in recent years, there’s been far more conversation with the Department of Medicine than there has with rehab” (P7).

Further, our findings highlighted the collaboration between the hospital and medicine as hospitals are given the opportunity to be involved in developing medical curriculum:

*[M]edicine…is way more ahead of…the other faculties in terms of co-creating curriculum and that comes from this structure where the people who are sitting at both tables are able to actually -- they're part of the same group.* (P5)

Despite these perceptions of unequal distribution of education resources, there were also noted strengths and opportunities as a result of medical education’s presence: “I think [rehab] could learn a little bit from what medicine is doing in terms of the actual give and take” (P3).

### Values and perceptions held by hospitals

Participants frequently discussed the impact of hospital values and perceptions on AHSPs. Participants experienced an inconsistency across facilities with regards to the symbiotic nature of the partnership:

*It’s not always a strong partnership because, in my mind, we’re working together, it’s a give and take, we’re getting as much out of it as we’re putting into it and I’m not sure that’s always the case with all the faculties at [the university]*. (P3)

Despite a pervasive perception that the university receives more tangible benefit from the partnership compared to hospitals, participants identified that hospitals value the status and prestige that comes with being associated with the university. As a result, hospitals are recognized and identified worldwide as reputable centers for education and patient care:

*I think the hospital benefits from the university’s identity internationally as well. Nobody knows what [hospital] is but everyone knows what [the university] is so there’s kind of a synergy that’s very important there*. (P12)

Participants stated that one of the ways hospitals demonstrate their commitment to education is including it as a priority in their mission statements and mandate. This works to engrain education into the hospital’s culture and espoused values:

*It’s the culture that we take students. We work with [the university]. They’re our primary partner and it’s sort of an expectation that this is how we work and teaching is a part of that. Teaching and learning is part of our culture*. (P2)

### Engagement between the hospital and the university

Active engagement, which we defined as the actions to participate in connecting the university and the hospitals, was another key influence on the partnership. As part of engagement, collaboration, communication, and involvement in the curriculum were emphasized.

Participants reported variable levels of collaboration in the current state of the partnership. While some individuals highlighted how collaboration exists in the partnership, stating that **“**everything from continuing education and programs we develop together, we do a lot of collaborative projects with the university and then all the research” (P12), others thought of it as an area for improvement, as “a lot of great ideas get put on the table, [but] nothing ever really gets done. And so, it can be a little bit frustrating in terms of that" (P3).

Participants continually spoke to the importance of explicit communication as an area that needed improvement to further enhance collaboration within the partnership: “I think key ongoing regular communication is really, really important…so, keeping those connections in terms of formalized structures, like meetings, things like that" (P3). Location, to a lesser degree, came up as a contributing factor to the diminished communication between the university and hospitals. Specifically, participants highlighted face-to-face communication and emphasized the importance of the university “to come out and just build those relationships… having a conversation… to kind of put names and faces together, to bridge faculty with certain research interests, maybe with clinicians working in the hospital, so that there’s that interconnectedness” (P2).

Another prominent topic that emerged from participants was the need for improved involvement in the curriculum development at the university instead of being a passive recipient of university generated curriculum change: “We can ensure success of the program, if we help co-create the curriculum" (P5).

In addition, participants identified the importance of having strong support from the university for their frontline staff to advance clinical education. One individual noted that:

*[P]roviding support when students are struggling or when there’s a challenging situation, I think that’s helpful because that’s a reality and struggles will happen and so, it’s been very helpful and the faculty has been engaged to help problem solve that*. (P9)

### Individuals’ engagement

The level of motivation and engagement of individuals within the hospital was identified as another dimension within the partnership. Participants noted that having experienced trainees was a motivator as they can increase clinician productivity:

*People love having senior trainees around because you definitely increase your productivity. They can see a patient when you're seeing a patient then you have a quick review and you would have taken twice as long yourself personally to see the patient*. (P5)

In contrast, taking on students was found to hinder clinician time management, and thus involvement in clinical education. One participant expressed that there are “incredible demands placed on clinicians… having to do more with less, all the time, makes it very challenging to have the time to be with a student” (P6).

Participants also commented on factors which facilitated clinicians taking on students. One such factor is the motivation of clinicians to participate in clinical education regularly, stemming from intrinsic desires to participate:

*The sort of feel good altruistic aspect…it's a rewarding experience oftentimes for staff and just the relationship when you get a great student… that's super satisfying. It's lovely*. (P2)

Intrinsic motivation was not the only reason for facilitators to taking on students as several participants brought up the importance of non-monetary recognition or acknowledgement of work put forth by clinical educators:

*So it doesn't come with money necessarily…you get a plaque, or you get a thank you note…so people who teach on behalf of the [hospital] need to be recognized at the [hospital] as well as locally*. (P5)

### Reciprocal opportunities

Reciprocal opportunities, which we defined as the products coming from the partnership with potential benefits for both academia and healthcare, further influence the partnership. Participants highlighted that the partnership with the university offers individuals access to research, with one participant stating:

*[M]any of the benefits come in terms of our access to resources, so, access to the library if you are faculty…which is really important because it allows us to access journals we can't necessarily get at some of our local libraries*. (P3)

Research access also included the universities’ access to research opportunities as well as the hospitals’ work opportunities and manpower to conduct research projects: “getting the students to participate helps us get research done and provides the actual human resource to do a lot of that work” (P9).

While participants praised research opportunities, they also discussed the importance of career opportunities for staff and highlighted the importance that a university partnership has in this regard:

*It allows us access to a community of practice…you really do get information about what's happening at the school, you stay in touch with different faculty…it gives the front-line clinicians an opportunity to potentially develop a career track*. (P3)

Participants also spoke to the hospital being afforded the opportunity to stay up to date with best practices as practitioners who participate in clinical education are driven by students to remain current in their field:

*I truly believe that having an academic partnership raises your quality bar, raises your ability to deliver the most advanced care and you have people who are coming in all the time who are very current, asking questions, very inquisitive, it creates a real environment of critical inquiry, of sharing, of advancing care. People are more open to change, take more risks, more open to redesign, so I think there’s only positive benefits for both patients and families, and also the staff and physicians*. (P3)

The ability of a hospital to be up-to-date and provide students with the most current health-care education and techniques was shown to enhance patient care: “The experience of the patients is far better because [they have] trainees who are interested in them, motivated to see them, and be part of their care team” (P14). Another participant voiced that:

*[H]aving an educational institution partnership actually increases knowledge translation across the board and ultimately benefits patient care because presumably you are providing the best possible up-to-date care*. (P5)

## Discussion

The primary goal of this study was to gain an in-depth understanding of the experiences of hospital leaders within AHSPs to contribute knowledge and understanding to support symbiotic partnerships. Ultimately, participants identified six dimensions shaping AHSPs. We found that these dimension of infrastructure within Health Science Education and the relationship and model of medical education delivery in relation to other professions shape the perceptions and values of the leaders in affiliated hospitals. These perceptions influence engagement between the hospital and the university as well as individuals’ engagement. It is through this engagement that there are emergent reciprocal opportunities from the university and hospitals joining to form an AHSP.

Creating and sustaining successful AHSPs requires understanding the complexities within the partnership, including the differences between each party. Pierre Bourdieu’s theory of practice provides a useful lens which helps to further explain, and expand upon, the dimensions we found to be shaping the partnerships. Bourdieu outlined how agents (either individuals or groups) shape the world they live in through their motivations and behaviours, and how social structure influences individuals.^21^ Further, Bourdieu explains how human motivations are driven towards gaining capital; which is any physical or social valued entity. His theory provides a useful way of viewing and re-thinking social practices and processes between different social fields (e.g., the hospital/healthcare and university/higher education fields). Finally, Bourdieu suggests it is only through becoming aware of the systems at play that we can shape them, offering implications for change.^21^

The AHSPs between universities and hospitals involve two separate fields coming together: education and healthcare. As each field is a separate entity, each field has its own rules inherently ingrained within the agents of that field.^21^ This is seen within our findings within the central dimension of values and perceptions of hospital leaders. Human perceptions and values, amongst many other things, are shaped by past experiences, such as education delivery models, and the environment, or infrastructure, in which we grew up and exist in,^22^ influencing the inherently ingrained rules. Hospital leaders highlighted that these rules influence individuals’ motivations. The largest influence of departmental values and culture lies with the institution itself, determining individuals within that fields’ perceptions of students, research, and professional workload and responsibilities.^23^ These inherently ingrained rules also dictate what capital, gained through hospitals partnering with the university, isvalued.^24^ Hospital leaders highlighted the status afforded by affiliation with the university as a key motivation for hospitals to partner; they benefitted from the symbolic capital of partnership. Further, the collaborative networks, support, and mentorship from the university offer a gain in social capital for the hospitals, while research collaboration and continuing education opportunities provided to the hospitals through the partnership act to increase their institutionalized cultural capital. As per Bourdieu, these forms of capital can be converted into other forms, including economic capital; for instance, increased research capacity affords increased funding potential for the hospital.^25^ This overall gain in capital will benefit the hospital, positioning it more advantageously in the overall healthcare social field. From this view, the partnership can be seen as a way for hospitals to acquire or increase certain forms of capital that the university affords, driving their motivation to engage in clinical education.

In a study by Brosnan,^26^ the existence of competition between universities in the field of medicine was highlighted. Their drive for capital is underpinned by the desire to generate high quality research, attract students with the highest grades, and generate high student satisfaction, which in turn increases capital held by the institution. Health science education students value the clinical placement opportunities they gain in the hospital setting and anticipate the inevitability of hospital-based clinical education as it is a central component to their education.^27^ As this is how medical education has always been delivered, it will continue to feed forward and reinforce itself through the habitual regeneration of the ingrained rules within academia.^28^ Not only are hospital placements integral to the university’s medical education, they also provide increased student satisfaction and therefore opportunity for student enrollment, which ultimately increases capital within the university. Thus, the partnership provides the opportunity for both the university and hospitals to gain coveted capital, positioning them more advantageously within the academic and healthcare fields, respectively.

As agents from the healthcare and academic field each have their own ingrained understanding of the rules and valued capital of their respective field,^24^ we noted partnership differences and differing professional perceptions between fields.^29^ When evaluating partnerships between schools and community education agencies, Tett et al.^30^ found that sharing and having complementary purposes is integral to effective partnerships. While sharing written purposes through the partnership agreement is of high value, the value of soft-skills in creating shared purpose is imperative as well.^31^ Hospital leadership echoed this through the stated value of the central concepts of communication, collaboration, and involvement within the university and hospital engagement dimension of the partnership.

Within any partnership, clear communication is key to successful collaboration,^32^ including many different forms and modes of communication. Frequent and two-way communication is essential to thoroughly conveying each parties’ respective purpose, philosophies and structures,^33^ including the valued capital within each respective field.^34^ Valuable and effective communication can take place in a variety of forms, however research supports the value of face-to-face communication surpassing all other forms with regards to comprehension and effectiveness.^35^ Nevertheless, current research shows that so long as there is trust-building, routinecommunication, media richness, and accountability, virtual communication is a successful alternative mode of communication.^36^ In addition, literature identifies regular communication to be an integral factor on strengthening the relationship between the university and their affiliated hospitals.^33^ Given the inherent differences between constituents of the healthcare and academic field, regular contact will help to enable understanding and a strong, symbiotic relationship. In our sample, regular contact between the university and hospitals was valued and led to engagement and reciprocal opportunities.

However, effective communication mechanisms and skills will not suffice if the dedication and motivation of all the members within a partnership is lacking. As per Bourdieu, motivation stems from the inherent drive to gain valued capital,^24^ which includes valued sources of recognition.^37^ While recognition is regularly thought to be monetary, non-monetary acknowledgement is often valued more.^38^ The participants of this study identified the value of non-monetary recognition as a key driver for involvement in clinical education. In this regard, the university could drive motivation and thus collaboration with public acknowledgement, plaques, certificates, and status appointments within the university. Further, the social capital via increased status amongst their peers, gained from recognition received from a prestigious institution such as the university, should stand to drive further participation within clinical education. Specifically, the literature identifies that individuals will sacrifice potential or real economic gain (i.e., time and money), in exchange for such status.^38^ This pursuit of status and social capital is often seen through clinician motivation to become involved with the university.

When individuals are involved in an organization, they feel more responsibility towards the mandates and thus will work harder to put such into action.^39^ While constituents have a strong responsibility towards their own respective field, being involved in interactions with the other field can drive understanding and responsibility towards a common purpose.^31^ Involvement of both parties in the creation of the shared curriculum of the health professions would also aid in fluidity of education delivery, helping standardize the delivery across hospitals.^1^ Universities may thus be advised to involve academic hospital personnel through joint curriculum design and having hospital staff represented on university committees. Their physical presence in the room will aid in their ability to have a voice and be a member of the conversations, engendering shared understanding of the academic health sciences field, and perhaps merging the differing fields into a true partnership.

This study successfully sought heterogeneity in the domains of community versus fully affiliated hospitals and current level of engagement with the university, however we were unable to obtain an equal gender split. Nonetheless, the sample gender ratio was similar to that found in the literature of 74.4% of PTs in Canada being women.^40^ Further, we successfully obtained our goal of three participants from the leadership levels of CEO, VP of Education, UPAR Committee Member and CCCE/PPL, however we were only able to recruit one VP of Practice. To mitigate the effects of this limitation, we purposively sampled from related levels of leadership to fill this vacancy (i.e., VP of Education). As potential participants were contacted based on purposive sampling, we were reliant on them consenting to be a part of our study, which could have led to inherent volunteer bias. Another potential limitation of our study was having two interviewers. Although the two interviewers both underwent the same training, the delivery and content of these probing questions could have influenced the responses of the participants. In addition, the perspectives obtained in our study strictly related to a partnership with the university and its affiliated hospitals. This is not reflective of the structure of all existing clinical education partnerships. Future studies would benefit from exploring perspectives of different organizations involved in educational partnerships across Canada, as well as exploring and comparing the perspective of the universities in addition to the hospitals.

### Conclusion

With a better understanding of hospital leadership’s perspectives on AHSPs, including what contributes to a successful partnership and what the facilitators and barriers are to their involvement, we made recommendations to support the best symbiotic and sustainable partnerships between universities and affiliated teaching hospitals. Based on our results, current literature, and the interpretation of our findings with support from Bourdieu’s theory of practice, our study suggests that the exchange of capital between hospitals and universities holds potential to be reciprocally beneficial. Therefore, communication, collaboration, and involvement of hospital partners in curriculum planning serve as modifiable components that may optimize partnerships. Ultimately, both fields stand to benefit from partnering to prepare the next generation of healthcare professionals.

## Appendix A

**Interview Guide**

To leadership: As outlined, we will be recording the interview in order to transcribe and analyze the points we discuss. I would like to reassure you that this interview will remain entirely confidential, and immediately after we finish here today, ____ and I will return to University of Toronto to transfer the audio file to an encrypted USB which will be secured under lock and key, and the audio recording will then be deleted from the audiorecorder.

Throughout this interview I would like you to share your perspectives on the partnership between the University of Toronto and this institution. When I say partnership, I am referring to your hospital’s agreement to partner with the university to educate healthcare students through clinical placements. I have a few questions to help guide our discussion but please feel free to elaborate on any area you feel is important and provide any information that my questions may not capture. Please remember you can share as much or a little as you would like.

1. Can you tell me what you know about the partnership that < hospital > has with the University of Toronto?
  - How does your role affect the institution's accomplishments?
  - What are you most proud of in regards to education in your facility?
  - Importance that hospital places on partnership?
  - If appropriate- Do you see any differences between your partnership with University of Toronto and other universities or colleges?
2. What do you think the benefits or the positive aspects of the partnership are?
  - In what ways does the partnership benefit you personally?
  - What kind of benefits does the partnership offer to the organization?
3. What do you think facilitates or enables these partnerships?
  - What aspects of the establishment facilitate the partnership?
  - Do you think that this partnership is sustainable in the long term?
  - What are the facilitators to sustain a successful partnership?
  - How do you think these partnerships benefit patient care?
  - What is University of Toronto doing that facilitates these partnerships?
  - What could the University of Toronto do more of or implement to strengthen the partnership?
4. What are some areas within this partnership that you feel need improvement?
  - What challenges do you experience personally in your role?
  - What sort of institutional factors contribute to the challenges you face in your role?
  - Any contributing factors from the healthcare system as a whole?
  - I hear that a lot of your examples pertain to (insert health discipline name here), what of what you are telling me do you think pertains to physical therapy (or other health disciplines)?
  - Is there anything we have discussed about clinical placements as a whole that you feel does not apply to physical therapy?
5. How do you feel being involved in the education of future healthcare professionals influences your frontline care providers?
  - Research indicates that management feels taking students decreases clinician productivity; workload measurement data indicates that productivity increases when clinicians take on students… Do you find this is a perspective that is shared among staff and/or management at your institution?
  - Are you aware that University of Toronto offers some compensation to the hospital for taking on students?
6. Thinking about our conversation, what would a great partnership between the University of Toronto and your institution look like to you?
7. Aside from what we have already discussed, is there anything you would like to add in?

Thank you very much for taking the time to share your valuable insight and perspectives: you have brought up many excellent ideas. Should you have any questions about the research project or about our meeting here today at any point, please don’t hesitate to contact myself, (facilitator present) or any other member of the research team. As our project moves forward over the next few months and our interviews have been analyzed, we will update you about the study’s progress and share our final report with you.
